# Inhibitory control effectiveness can be improved: The role of arousal, subjective significance and origin of words in modified Emotional Stroop Test

**DOI:** 10.1371/journal.pone.0270558

**Published:** 2022-06-28

**Authors:** Kamil K. Imbir, Joanna Duda-Goławska, Maciej Pastwa, Adam Sobieszek, Adrianna Wielgopolan, Marta Jankowska, Aleksandra Modzelewska, Jarosław Żygierewicz

**Affiliations:** 1 Faculty of Psychology, University of Warsaw, Warsaw, Poland; 2 Biomedical Physics Division, Institute of Experimental Physics, Faculty of Physics, University of Warsaw, Warsaw, Poland; Yamaguchi University: Yamaguchi Daigaku, JAPAN

## Abstract

The interference control measured in the Emotional Stroop Task is the phenomenon that gives us an insight into mechanisms of emotion-cognition interactions. Especially the role of dimensions of affect can be easily studied with this paradigm. In the current study, we were interested in the role of the complexity of emotional stimuli (origin). We also aimed at searching for activation-like factors that impair (arousal) or improve (subjective significance) the effectiveness of cognitive control. We have used an orthogonal manipulation of all the above dimensions in words. We expected to find the contrastive effects of arousal and subjective significance on reaction times and Event Related Potential’s amplitudes. On a behavioural level, we observed the reduction of reaction times with increasing subjective significance of stimuli and reflective origin. We also found a correlation between subjective significance and reduction of amplitude polarisation in the N450 component associated with cognitive control execution effort. This experiment shows that subjective significance has an improving role for cognitive control effectiveness, even when valence, arousal and origin levels are controlled. This guides us to conclude that external stimuli may drive not only disruption of control but also its improvement.

## Cognitive control in Emotional Stroop Tests (EST)

Cognitive control is a crucial mechanism of mental ability [1]. The capacity to control activities and plan them in relation to targets and expectations is a vital factor in the cultural development of humankind [2]. Furthermore, this ability to orchestrate our actions and cognitions in accordance with internal goals allows us to develop new situations [3] and to behave in a way leading us to achieve our aims [4, 5], instead of following automatic responses and attentional biases.

Cognitive control is a complex process that involves many independent (though partially correlated and related) functions [6, 7]. Among them is *updating*, which is based on refreshing the information in working memory, and then changing and monitoring its contents; *shifting*, which is the control of transferring between processes (switching) and maintaining the operating pattern while performing the second task; and *inhibition*, which is a suppression of an automated reaction or interference [6–8].

An ability to inhibit inaccurate responses or cognitions is called interference control, which allows us to resolve the conflict between task-relevant and task-irrelevant stimulus or its features. Thanks to interference control, we can suppress the competitive stimulus (due to the working memory function—*cognitive inhibition*) and also an automatic reaction (by motor control—*response inhibition*), and we can reduce the impact of this conflict on performance [4, 6].

Among the available paradigms to measure the interference control component of cognitive control, the Stroop task [9] is the most commonly used. The Stroop task allows us to detect the interference between the automatic and controlled processes. Furthermore, in the classical Stroop task, the visual characteristics of the stimuli (ink colour) and the semantic meaning of the presented word match in the case of congruent trials (e.g., the word “green” is written in green coloured ink), in contrast to the incongruent trial, where the word’s colour and meaning are divergent (e.g., the word “green” is written in red ink), so in this paradigm two different mechanisms occur. In the congruent trials, an automatic, well-trained, and often used reading process is sufficient to solve the task. Consequently, answers are provided quickly and correctly. In an incongruent trial, the visual and semantic aspects of stimuli are not consistent, which makes the task more cognitively demanding. The automatic processes that induce participants to read and decode the meaning of a word must be suppressed, and instead they have to focus on the font colour of the word. It is therefore necessary to suppress the automatic process to accomplish this task properly. Thanks to interference control, it is possible to resolve this cognitive conflict and reduce its impact on the task’s performance [4, 6, 10].

Researchers have pointed out that this mechanism is sensitive to the affective state of the participant. Affect influences the effectiveness of maintaining control over distracting, unwanted or task-irrelevant stimuli [11, 12]. It is considered that emotions influence attention processes and they play an important role in resolving cognitive conflicts [13, 14]. Therefore, the Emotional Stroop Task (EST) is a useful tool that considers the impact of emotions on cognitive processes. Previous studies that have used this paradigm have confirmed that the emotional qualities of the stimulus, which are not even related to the task, impact its performance [15].

As in the original paradigm (i.e., the Stroop Task), in the EST the participants are asked to name the font colour in which the verbal stimuli are displayed. However, unlike the classic task, these stimuli are not colour names but different words. Some of these words have an emotional load, while some of them are perceived as neutral (e.g., "table"). Therefore, there are no analogies here concerning congruent and incongruent variants, and the facilitation and inhibition processes related to them, respectively. In the EST, the word "sink" printed in blue is neither more nor less congruent than the word "cup" printed with yellow letters. The incongruence arises from the fact that the meaning of words has an emotional charge. This feature of the stimulus influences the processes of attention and the task’s performance [15, 16].

### Emotional factors influencing the EST

There are several approaches to the structure of affective states. It is possible to focus more on categorising emotions and then analysing them as a whole monolithic state (see, for example, [17]). However, another approach analyses emotions based on some of the dimensions underlying them (e.g., valence, arousal, origin and subjective significance), which shape and define the very nature of a particular emotional experience [18, 19]. The first dimension that is important for the EST performance is the valence representing the pleasantness versus the unpleasantness of an emotional experience [18]. However, valence effects in the EST have been explained by the arousal differences [4, 15, 16]. Therefore, in the current study, we have decided to exclude the valence and only control it among our stimuli by selecting those that are neutral in valence.

The first dimension that we have distinguished in the present study is the dual origin of an affective state; that is, automatic versus reflective [19, 20]. This dimension determines whether the emotion was created due to automatic (fast, effortless) or controlled (slow, effortful) processes [21, 22]. Metaphorically, it may be associated with emotions arising from the heart or mind [20, 23, 24]. Heart means an immediate and impulsive reaction to external stimuli, which should be linked to primal, intuitive thinking [22]. In contrast, the mind is related to much more deliberative, reflective thinking [25], which usually involves assessing the situation and comparing it with some existing standards [19].

Another dimension—the first of two dimensions of activation which we decided to study—that is considered to be fundamental for every emotion is arousal, which can be defined as the level of energy or the activity experienced during affective states [18, 26]. Emotional arousal allows us to engage in some action and react appropriately to the situation and it is an automatic, biologically programmed form of activation [27]. Finally, we took into account the reflective form of an activation mechanism that is called subjective significance, which is here understood as an attitude towards some stimuli or situation [2]. The subjective significance of particular objects may vary in time [28], depending on how these objects are interpreted and how they correspond with the current goals of an individual [2]. Some previous experiments involving the EST procedure experienced the effects of the interaction of two dimensions: arousal and subjective significance. Namely, words of high arousal and moderate subjective significance had significantly longer reaction times (RTs) when compared to words of moderate arousal and moderate significance, high arousal and low significance, and high arousal and high significance [2].

### EEG components in EST

We assume that in a well-designed experiment, the stimuli and task being performed by a participant evoke a characteristic sequence of neural activations corresponding to underlying mental processes. These activations are immersed in the ongoing brain activity (an additive model). In a single trial, the contribution from the neural populations engaged in the task to the amplitude of the Event Related Potentials’ (ERPs) deflection depends on the size of the neural population and the level of its synchronisation [29]. In the averaged ERP, additionally, the latency jitter matters [30]. Such contributions from well-defined neural ensembles we call latent components. However, we must be aware that the identical shape of ERP measured on the scalp can be obtained by summing up the contributions of many different hypothetical latent components. Therefore, comparing the amplitude of deflections in the ERP curve is reasonable only in well-defined, analogous tasks. Furthermore, identifying an ERP deflection as a component reported in the literature requires considering its topography, latency and experimental conditions.

The distraction caused by emotionally laden words during the EST may also be observed in certain Event-Related Potentials. These differences have been observed during a time course of the task, from early components, such as P2 and Early Posterior Negativity (EPN), to later components, such as P300 and N450 [31–33].

The P2 component may be observed in the time range of 200–250 ms from the stimulus onset, originating from the centro-frontal and occipito-parietal regions [34]. The differences between emotionally laden words and neutral words have frequently been observed within this component [35–37], with emotional words evoking higher amplitudes than neutral words. This effect may be interpreted as the difference between words that are high and low on arousal. This has been confirmed in a study that outright controls emotional arousal [27], where amplitudes were also more positive for highly arousing words. In the same study, the effect of subjective significance was observed in a time window that is related to the P2 component, with larger amplitudes for mildly significant words than for highly significant words.

In the same and following period of 200–300 ms after the stimulus onset, the EPN component may be observed over the occipital scalp [31]. This component has been considered to be an indicator of actions that are taken volitionally [31]. As in the P2, the differences in processing between emotional and non-emotional stimuli have also been observed here. In particular, the emotional stimuli evoke larger amplitudes, which may again be interpreted as high arousal of words influencing the processing [38–41].

In the following time window of 250–500 ms, the P300 component, which is a positive waveform over parietal areas of the scalp, can be observed [42]. This component is traditionally tied to categorising the stimuli, and identifying new or odd stimuli [43]. Some studies suggest that the emotionality of the stimuli may be influencing the amplitudes within the P300 component in the EST [37, 44]; as stated earlier, these effects may be tied to the arousing value of emotionality. Emotional stimuli have been reported in the time window corresponding to the P300 component effect of origin, with reflective stimuli evoking larger amplitudes than the automatic stimuli [45].

In the late sections of stimuli processing, a component called N450 has been identified as a negative-going waveform ranging from 350–500 ms, which is usually observed over the fronto-central areas of the scalp [46, 47]. This component is considered to be an indicator of conflicting stimuli, which is frequently reported in Stroop tasks and modified Stroop tasks [47–49]. Studies employing the EST procedure have suggested that emotional factors influence processing within this component, reporting differences in processing emotional and non-emotional stimuli [33, 46]. The effects of origin have also been observed within this component, with reflective-originated words evoking more positive amplitudes than automatic-originated words [33, 45].

### Aim and hypothesis

This study aims to investigate the role of three different affective dimensions of words—origin, arousal and subjective significance of affective reaction—in cognitive control efficiency as measured with a modified EST (performed for verbal stimuli neutral in valence). Our modification of the Emotional Stroop Task procedure lies in the use of neutral words (whilst the emotionality in the EST was usually derived from manipulating valence of the stimuli), however, our manipulation consists of stimuli differing on other emotional dimensions (origin, arousal and subjective significance). Therefore we consider it an emotional task. This is the first experiment to orthogonally cross these three dimensions; thus, the experiment was in part exploratory. Nevertheless, there are some instances of previous studies that have investigated these dimensions separately to formulate some predictions. In this study, we expected automatic origin to be more associated with primary emotions [21, 22, 24], which should lead to a disruption of control, while reflective origin should lead to an improvement of control. We also predict the increasing arousal level to disrupt cognitive control, while increasing the subjective significance level to cause an improvement in cognitive control efficiency, both in reaction times (i.e., shorter RTs for improvement of control) and in the N450 component susceptible to cognitive control amplitude (i.e., less negative amplitudes for improvement of control effectiveness).

## Materials and methods

### Participants

We conducted a priori analyses to estimate the sample size that would allow the study to achieve high statistical power. Basing our estimations on previous studies conducted in a similar paradigm [27, 50], we assumed that the *η*_*p*_^*2*^ for main effects of one factor could range from .05 to .15, and the interaction effects could achieve *η*_*p*_^*2*^ of .10. The a priori estimations using G-Power software [51] showed that to achieve high statistical power of .80 we would need at least 9 participants for main effects and at least 18 participants for interactions of two factors. These small sample sizes would be adequate due to the study design—a large number of repeated measures. Nevertheless, we decided to increase the sample to 36 participants in order to verify possible two-factor interactions with smaller effect sizes and identify possible three-way interactions.

The participants were recruited from various faculties at Warsaw’s universities. They had to meet the following criteria to be included in the experimental group: they had to be right-handed native Polish speakers, without chronic clinical issues that may affect EEG recording directly (e.g., epilepsy) or through medicine being taken because of a medical issue (e.g., anxiety). The participants’ vision had to be normal or corrected to normal. They received a small compensation for taking part in the experiment.

The entire experimental group consisted of 36 subjects (18 men and 18 women), who were aged from 19 to 36 years old (*M* = 24.47; *SD* = 4.5). After collecting the data, one participant was excluded from EEG analyses because more than 50% of her trials were rejected due to artefacts or extremely short or long response times. Therefore, 35 participants were included in the further analysis, 18 men and 17 women, who were aged 19 to 36 years (*M* = 24.6; *SD* = 4.24).

The participants provided their written informed consent to participate in the experiment. We did not collect any personal data that would allow the identification of the participants. The bioethical committee at the Faculty of Psychology, University of Warsaw, approved the design, experimental conditions and procedure. Moreover, all of the procedures involving human participants were done in accordance with the ethical standards of the institutional and/or national research committee, and with the 1964 Helsinki declaration and its later amendments or comparable ethical standards.

### Design

This study aimed to verify the behavioural and electrophysiological measures of the processing of emotionally charged words. This study’s design includes manipulation of three factors: valence (three levels), arousal (three levels) and subjective significance (three levels). The following properties of words were controlled: frequency of appearance in language and length. Two dependent variables were measured: (1) reaction latencies in the EST and (2) amplitudes of ERP for selected time windows or components.

### Materials

The words that we used in this study were obtained from the Affective Norms for Polish Words Reloaded (ANPW_R) database [52]. This database provides ratings for 4,900 Polish words on eight different affective measures, among which origin, arousal, subjective significance and valence were the dimensions of interest. When we established the database, each word was rated on each dimension by 50 participants (half of whom were men) on a Self-Assessment Manikin (SAM) scale. The ratings were then averaged, yielding means for every scale.

We constructed 27 groups (3 x 3 x 3 design, 15 words in each group, 405 words altogether), differing in levels of origin (automatic, null, reflective), arousal (low, medium, high) and subjective significance (low, medium, high), but not in any of the controlled factors. We controlled for valence, word length (number of letters) and frequency of use [53]. The words were chosen only among nouns, which constitute 59% of the original database. We decided to exclude all adjectives from our final selection because Polish adjectives differ in form according to the grammatical gender of the noun that they refer to. Possible issues stemming from this fact could not be controlled because the affective norms database that we used included only adjectives in their masculine form. It would also mean that each adjective would end on a very similar syllable, making the difference between nouns and adjectives phonologically obvious.

In our final list of 405 words, the mean ratings when divided by the three levels of origin were *M* = 4.65, *SD* = 0.38 for automatic, *M* = 5.46, *SD* = 0.28 for null and *M* = 6.41, *SD* = 0.40 for reflective stimuli. Mean ratings of words divided by arousal were: *M* = 3.38, *SD* = 0.32 for words with low arousal, *M* = 4.09, *SD* = 0.27 for moderately arousing words and *M* = 4.87, *SD* = 0.36 for highly arousing words. For the dimension of subjective significance, *M* = 3.12, *SD* = 0.33 for low, *M* = 3.81, *SD* = 0.27 for medium, and *M* = 4.80, *SD* = 0.42 for highly significant words.

To verify our selection, we performed a 3 (origin) × 3 (arousal) × 3 (subjective significance) ANOVA analysis for each of the six variables (three manipulated and three controlled), which we treated as dependent variables. We expected to find only three significant effects, namely an effect of arousal-on-arousal ratings, of origin-on-origin ratings and of significance-on-significance ratings if the selection was correct. The data for each word’s frequency of use was transformed into natural logarithms to more closely approximate a normal distribution.

For groups divided by origin, there were significant differences in origin ratings: *F*(2, 378) = 826.05, *p* < .001, *η*^*2*^ = .814, but not in arousal ratings: *F*(2, 378) = 1.19, *p* = .306, *η*^*2*^ = .006, or in ratings of subjective significance: *F*(2, 378) = 0.13, *p* = .882, *η*^*2*^ = .001. There were no differences between groups in terms of valence ratings: *F*(2, 378) = 1.38, *p* = .254, *η*^*2*^ = .007, the number of letters: *F*(2, 378) = 0.40, *p* = .668, *η*^*2*^ = .002, and there were no significant differences in frequency: *F*(2, 378) = 0.37, *p* = .692, *η*^*2*^ = .002.

We found significant differences in arousal ratings between groups divided by arousal: *F*(2, 378) = 725.74, *p* < .001, *η*^*2*^ = .793, but not in ratings of subjective significance: *F*(2, 378) = 0.24, *p* = .786, *η*^*2*^ = .001, nor origin: *F*(2, 378) = 1.08, *p* = .339, *η*^*2*^ = .006. For controlled dimensions, there were no differences between groups of different arousal in terms of valence ratings: *F*(2, 378) = 2.66, *p* = .071, *η*^*2*^ = .014, or for terms of word length: *F*(2, 378) = 0.65, *p* = .525, *η*^*2*^ = .003 or frequency of use: *F*(2, 378) = 1.56, *p* = .212, *η*^*2*^ = .008

Finally, for groups divided by subjective significance, we found differences in subjective significance ratings: *F*(2, 378) = 807.10, *p* < .001, *η*^*2*^ = .810. However, the groups did not differ in arousal ratings: *F*(2, 378) = 0.72, *p* = .488, *η*^*2*^ = .004 or ratings of origin: *F*(2, 378) = 0.39, *p* = .680, *η*^*2*^ = .002. There were also no differences in valence: *F*(2, 378) = 2.66, *p* = .071, *η*^*2*^ = .014, word length: *F*(2, 378) = 1.86, *p* = .158, *η*^*2*^ = .010 or frequency of use: *F*(2, 378) = 1.31, *p* = .271, *η*^*2*^ = .007. The analyses also showed no significant interaction effects because we did not find that any of the three possible two-way interactions between manipulated factors (arousal and subjective significance, origin and subjective significance and origin and arousal) were statistically significant, either for manipulated or controlled dimensions. In addition, there was no three-way interaction between origin, arousal and subjective significance on any of the manipulated or controlled scales. All of the 405 words that we used in the experiment, as well as their affective measures, length and frequency of use, can be found in Appendix 1; we also present some examples of words differing in their origin, arousal and subjective significance levels in Table 1.

**Table 1:** Some examples of words differing in their origin, arousal and subjective significance levels; the full list of words is presented in S1 Appendix.

Origin			Arousal			Subjective significance		
Automatic	Null	Reflective	Low	Medium	High	Low	Medium	High
angel	beetle	emperor	corridor	rose	dancer	flower	mirror	fatigue
cloud	part	chalice	matrix	dog	tiger	tingle	moon	religion
calf	canal	robot	box	magic	sweat	cat	flame	freedom

### Procedure

The participants were invited to an EEG lab. They sat in a comfortable chair in front of a 15.6 inch LCD screen located approximately 1 m from their eyes. The procedure involved a simultaneous presentation of the target word and the cue indicating possible responses (letters: P, C, Z, N) below the target word of the screen. The font used in the study was Helvetica 50 point. The first part of the experiment included a training session to prepare the participants for the main session. There were 20 initial trials during which the participants were asked to name the squares’ colour (there were four target colours) and to read a colour-meaning word. The training session was followed by 60 trials of the standard Stroop test [9]. The participants named the font colour of the target word (ie. blue, red, green, yellow). The words were randomly either displayed in a congruent or incongruent manner. The instructions included suggestions to respond as quickly and accurately as possible.

The next stage of the experiment involved indication of the font colour of emotionally involving words. At the beginning, a fixation cross was presented in the middle of the screen for 700 ms. Subsequently, the target word was presented. The time of word presentation was determined by the time of the subject’s reading and responding; however, the minimal exposition duration was 300 ms. Afterwards, a blank screen appears for 700–800 ms.

The words were presented in groups of 15 words of the same affective characteristic. A block design in the EST is advised by the previous research because it enables us to obtain more noticeable effects [54] After each group presentation, a short 3 s break was available for participants.

In total, there were 27 groups. The group division reflects all of the possible combinations of three factors at three levels (3 origin x 3 arousal x 3 subjective significance), consisting of a list of 27 groups comprising 405 words (27 x 15 words).

**Fig 1:**
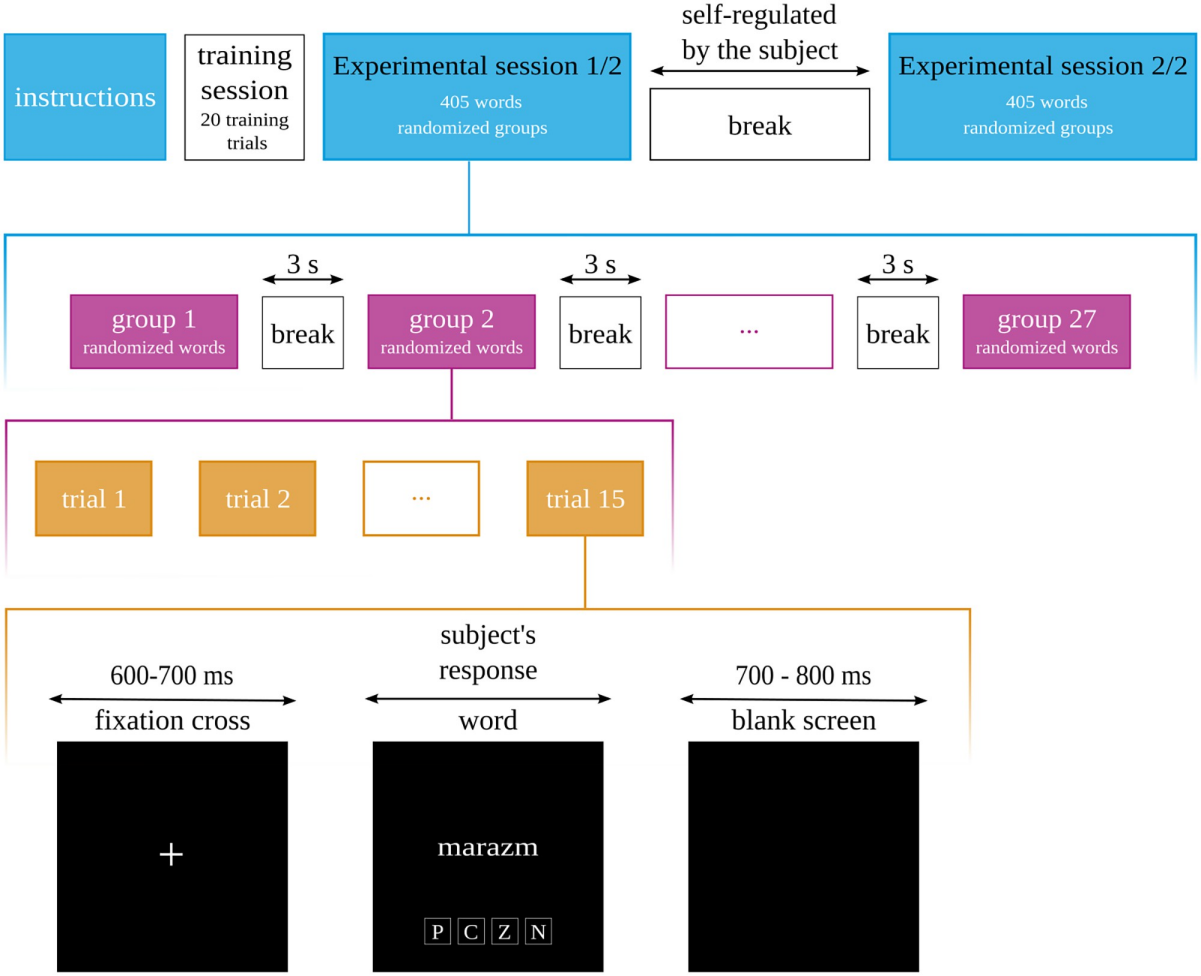
Outline of the experimental procedure.

Both groups and words in each group were presented in random order. The experimental session was built of two separate parts, with a break between each. Each participant could decide how much time they wanted to spend on the break. The experiment’s procedure is shown in Fig 1.

### EEG recording and offline data processing

The stimuli were presented on a 15.6-inch LCD screen. The stimuli were synchronised to EEG data by a signal from a circuit recording the brightness of a small rectangle that changed synchronously with the screen’s content. The rectangle was hidden from the subject’s view.

We recorded EEG from a standard 10–20 system (i.e., Fz, Cz, Pz, Fp1/2, F7/8, F3/4, T7/8, C3/4, P7/P8, P3/4 and O1/2) referenced to linked earlobes and grounded at the AFz position. All impedances were kept at a similar value below 5 kOhm. The signals were sampled at 1024 Hz using a Porti7 (TMSI) amplifier.

Offline signal processing was done with Matlab^®^ using EEGLAB toolbox [55] and custom-made scripts. The signal was zero-phase filtered using second-order Butterworth filters (12 dB/octave). The high-pass cutoff was 0.1Hz, and the low-pass cutoff was 30 Hz. Additionally, we used a notch filter (second-order Butterworth with stop-band 49.5–50.5 Hz).

We extracted epochs from -200–800 ms, with 0 being the target stimulus’s onset. The signals were baseline corrected (-200–0 ms). We removed trials with a logarithm of the response time (RT) shorter than the (Q1—W) or longer than the (Q3 + W), where Q1 and Q3 are the 25th and the 75th percentile of the individual distribution of ln(RT) for each subject, W = 1.5*(Q3 − Q1). Effectively, the response time is within 298–4422 ms for the analysed data across all subjects; the percentage of trials removed due to RT trimming was 2.28%. Additionally, we removed trials in which the subject wrongly identified the presented word’s colour from further analysis (2.21%). The remaining trials (*M* = 28.65, *SEM* = 0.04 per condition) were used to analyse behavioural variables. Finally, we checked the uniformity of reaction accuracy for all pairs of factors using the Friedman test for replicated block design and did not obtain any significant differences.

For the analysis of ERPs, we additionally removed trials with eye blinks (21.7%), getting on average (*M* = 23.5, *SEM* = 0.1) trials per condition. We checked the uniformity of the number of trials per condition for all pairs of factors using the Friedman test for replicated block design and did not obtain any significant differences.

### Statistical analysis

For the distribution of variables, the response accuracy and the number of correct and artefact-free trials were not normal. Therefore, the significance of these variables’ effects was assessed using the Friedman test for replicated block design.

Reaction time and EEG effects were analysed using a three-way ANOVA with repeated measures. In the case of reaction times, we used logarithm transform to render the distribution normal. The transformed reaction time was the dependent variable, and the independent variables were origin, arousal and subjective significance. The significant main effects were analysed with post-hoc paired t-tests with Holm’s correction for repeated comparison [56].

The scheme of analysis of the classical EEG components was analogous to that used for the behavioural data. The dependent variable was the mean amplitude of a component (averaged across the electrodes and time range proper for the component). The independent variables were origin, arousal and significance. Sphericity was checked with Mauchly’s test and we applied Greenhouse-Geisser correction where necessary. The significant main effects were analysed with post-hoc paired t-tests with Holm’s correction for repeated comparison [56]. The significant interaction effects were further investigated with a series of one-way ANOVAs, with the levels of a chosen variable set iteratively to subsequent levels. The significance of the effects in the series of ANOVAs was corrected for multiple comparisons by Bonferroni correction. The observed significant main effects were further investigated using post-hoc t-tests with Holm’s correction. In this study, we did not obtain significant three-way interactions.

The procedures were implemented in the R statistical package [57].

## Results

### Behavioural results

**Fig 2:**
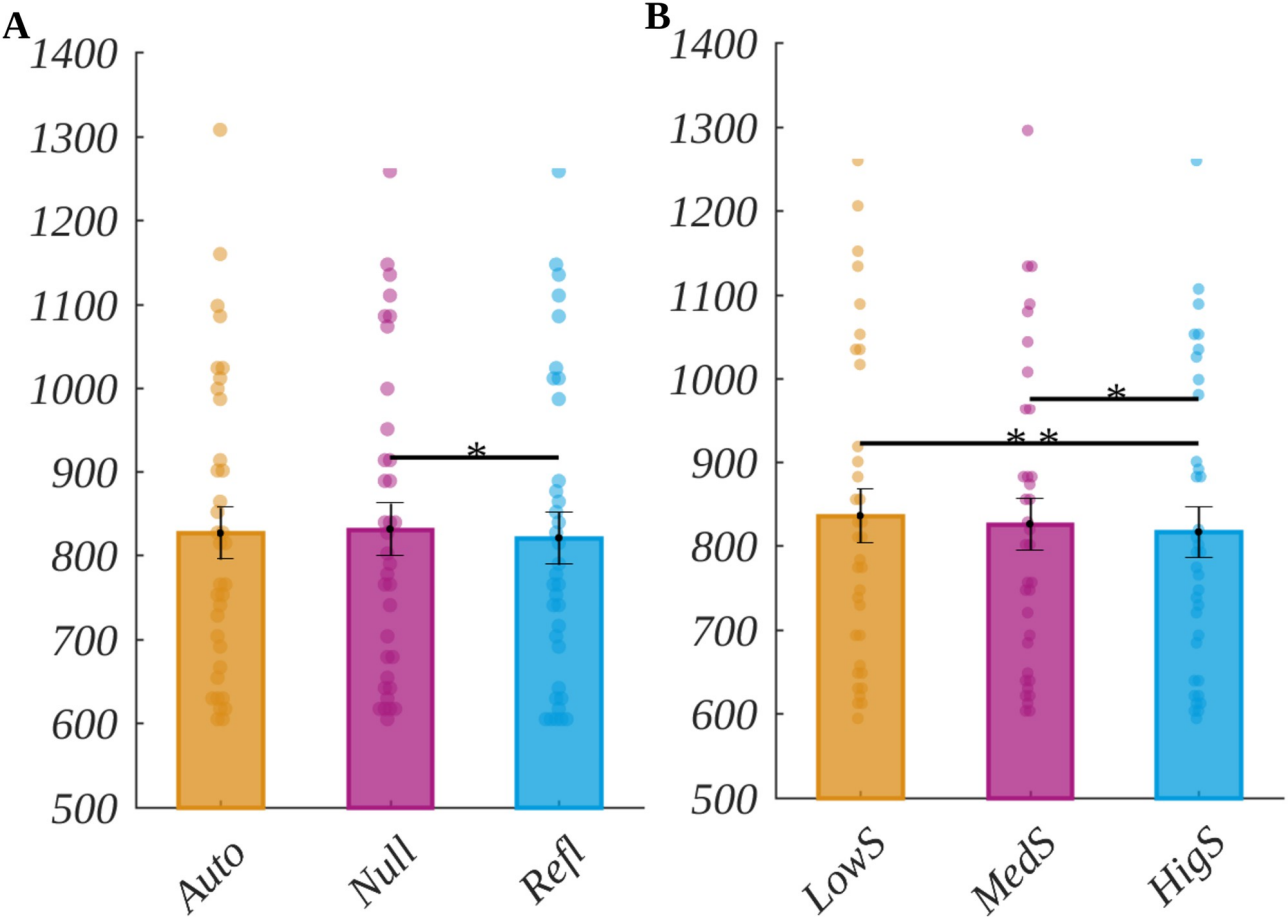
Main effects in reaction times for: A origin, B subjective significance. The bars represent the mean value, error bars SEM, individual dots mark the average response time of an individual subject in the given condition, the black horizontal lines indicate pairs of conditions with significantly different means (** *p* < .01, * *p* < .05). Response time is given in ms.

We analysed the reaction times using repeated measures ANOVA. The natural logarithm to transform RT was the dependent variable, while origin, arousal and subjective significance (each with three levels) were the independent variables. The post-hoc tests were corrected with a Holme’s correction. The results are summarised in Fig 2. We obtained the main effect of origin (*F*(2, 68) = 3.55, *p* = .034, *η*_*p*_^*2*^ = 0.09). The post-hoc test showed that reaction time for reflective words (*M* = 820.62, *SEM* = 31.03) was significantly shorter than for the null words (*M* = 831.10, *SEM* = 31.18; *t*(34) = -2.85, *p* = .022, *d* = 0.98). Moreover, the main effect of subjective significance was also significant (*F*(2, 68) = 9.16, *p* < .001, *ηp*^*2*^ = 0.21). The post-hoc test revealed that the reaction time for highly significant words (*M* = 816.39, *SEM* = 29.95) was significantly shorter than for both low significant words (*M* = 835.69, *SEM* = 32.08; *t*(34) = -3.976, *p* < .001, *d* = 1.36) and for medium significant words (*M* = 825.91, *SEM* = 30.68; *t*(34) = -2.42, *p* = .04, *d* = 0.83). The main effect of arousal was not statistically significant (*F*(2, 68) = 0.037, *p* = .96).

### ERP component analysis

We performed a three-way ANOVA with repeated measures analysis for components that are typically susceptible to the emotional properties of emotion-laden words in EST (i.e., P2, EPN, P300 and N450). The dependent variable was the average amplitude in the appropriate time window and ROI for a given component. The independent variables were origin, arousal and subjective significance (each with three levels). Post-hoc tests were corrected with Holme’s correction.

**Fig 3:**
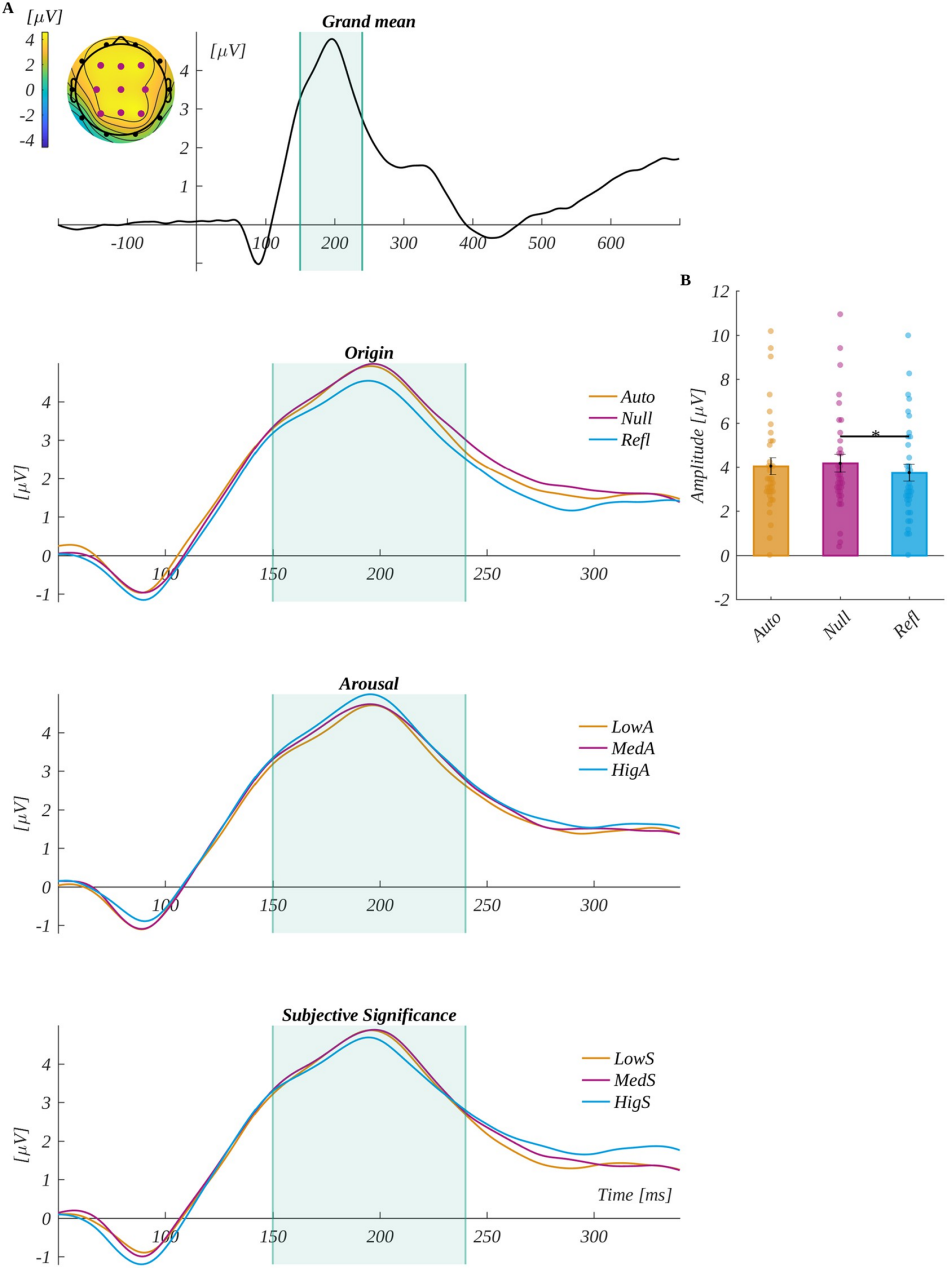
The P2 component results. The upper plot A is the grand average across electrodes from ROI_P2_. The marked time-interval was selected for the P2 analysis. The insert shows the topography of the mean potential from this period with the electrodes forming the ROI_P2_ marked with pink dots. The three bottom plots are the time courses of ERP averaged across electrodes from ROI_P2_ for each level of origin, arousal and subjective significance. Main effect for: B origin. The bars represent the mean value, error bars SEM, individual dots mark the average amplitude of an individual subject in the given condition, the black horizontal lines indicate pairs of conditions with significantly different means (* *p* < .05).

We analysed the P2 component in the characteristic region of interest (ROI_P2_) (i.e., F3, Fz, F4, C3, Cz, C4, P3, Pz and P4) in the time window of 150–240 ms. The average signals from the ROI_P2_ electrodes for each level of origin, arousal and subjective significance are shown in Fig 3A. We observed the main effect of origin (*F*(2, 68) = 6.13, *p* = .004, *ηp*^*2*^ = 0.15). The post-hoc tests showed that amplitude for reflective words (*M* = 3.74, *SEM* = 0.38) was significantly lower than in the null words (*M* = 4.17, *SEM* = 0.40; *t*(34) = -2.97, *p* = .016, *d* = 1.02). The effect is shown in Fig 3B.

**Fig 4:**
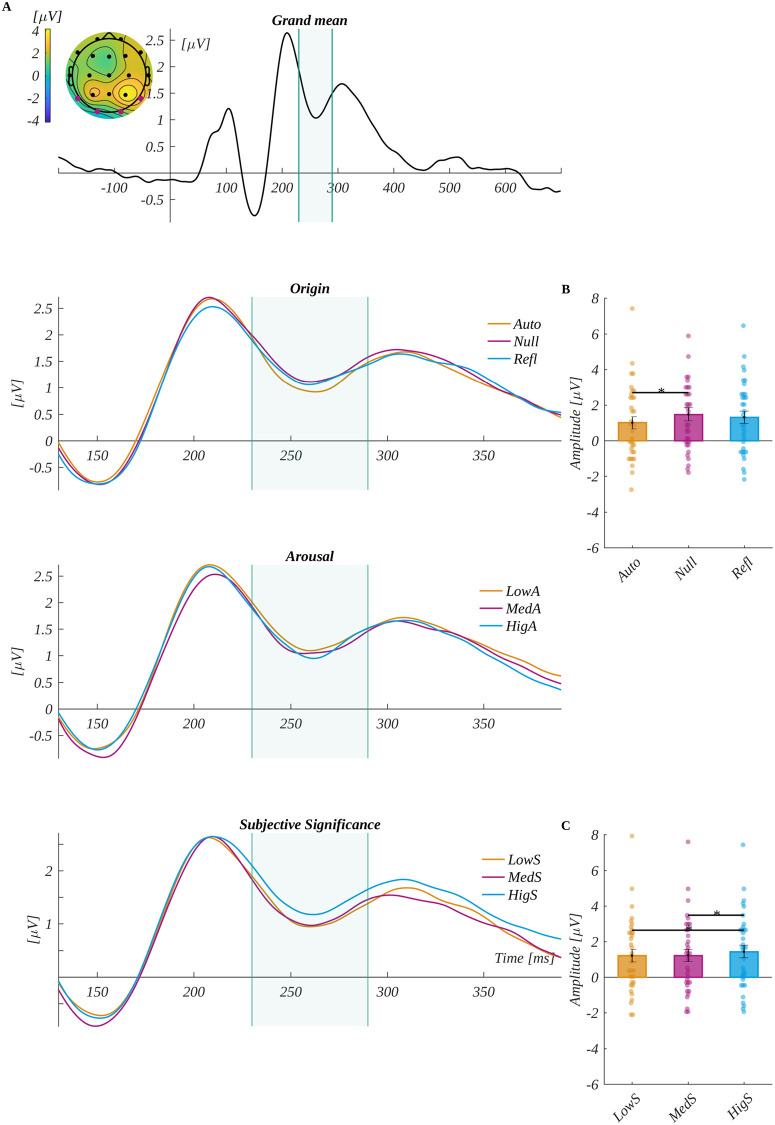
The EPN component results. The upper plot A is the grand average across electrodes from ROI_EPN_. The marked time-interval was selected for the EPN analysis. The insert shows the topography of the mean potential from this period with the electrodes forming the ROI_EPN_ marked with pink dots. The three bottom plots are the time courses of ERP averaged across electrodes from ROI_EPN_ for each level of origin, arousal and subjective significance. B Interaction between arousal and origin: differences between origin levels for medium arousal words. C Main effect for subjective significance. The bars represent the mean value, error bars SEM, individual dots mark the average amplitude of an individual subject in the given condition, the black horizontal lines indicate pairs of conditions with significantly different means (* *p* < .05).

The EPN component was analysed in the time window of 230–290 ms in its characteristic region of interest (ROI_EPN_) (i.e., O1, O2, T5 and T6). The traces obtained from ROI_EPN_ electrodes for each level, origin, arousal and subjective significance, and the grand mean are shown in Fig 4. We observed that the main effect of subjective significance (*F*(1.57, 53.33) = 5.81, *p* = .009, *η*_*p*_^*2*^ = 0.15) was found. Mauchly’s test indicated that the assumption of sphericity had been violated for this factor (χ^2^(2) = 0.72, *p* = .005), and therefore degrees of freedom were corrected using Greenhouse-Geisser estimates of sphericity (∈ = 0.78). The post-hoc tests revealed that amplitude for highly significant words (*M* = 1.44, *SEM* = 0.36) was more positive than for both low significant words (*M* = 1.20, *SEM* = 0.36; *t*(34) = 2.48, *p* = .037, *d* = 0.85) and medium significant words (*M* = 1.22, *SEM* = 0.36; *t*(34) = 3.05, *p* = .013, *d* = 1.05). Furthermore, the effect of an interaction between arousal and origin was found to be significant (*F*(4, 136) = 3.06, *p* = .019, *η*_*p*_^*2*^ = 0.08). In a series of ANOVA tests for each level of arousal, we obtained a significant effect of origin for medium arousing words (*F*(2, 68) = 5.845, *p* = .005, *η*_*p*_^*2*^ = 0.15). The post-hoc tests indicated that amplitude for null words (*M* = 1.48, *SEM* = 0.38) was significantly more positive than for the automatic words (*M* = 1.00, *SEM* = 0.35; *t*(34) = 3.14, *p* = .011, *d* = 1.08).

**Fig 5:**
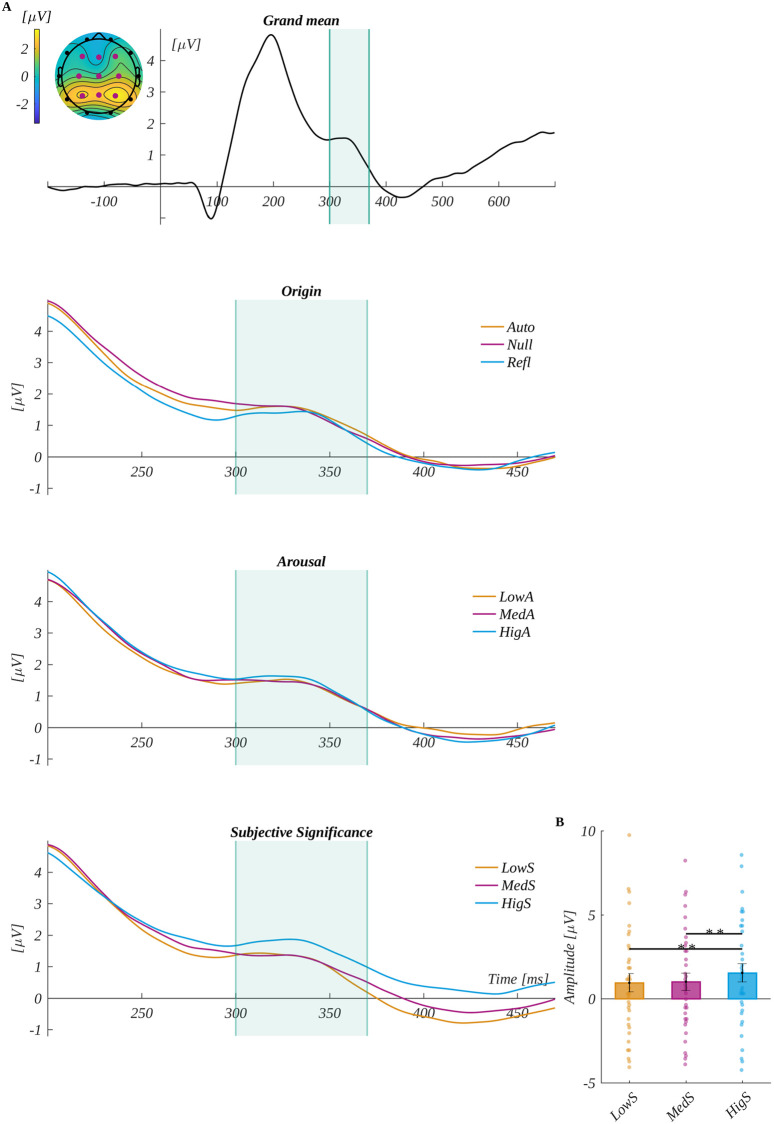
The P300 component results. The upper plot A is the grand average across electrodes from ROI P300. The marked time-interval was selected for the P300 analysis. The insert shows the topography of the mean potential from this period with the electrodes forming the ROI_P300_ marked with pink dots. The three bottom plots are the time courses of ERP averaged across electrodes from ROI_P300_ for each level of origin, arousal and subjective significance. B Main effect for subjective significance. The bars represent the mean value, error bars SEM, individual dots mark the average amplitude of an individual subject in the given condition, the black horizontal lines indicate pairs of conditions with significantly different means (* *p* < .05, ** *p* < .01).

We analysed the P300 component by averaging the ERP amplitude in the time window: 300 ms to 370 ms in the characteristic region of interest (ROI_P300_) (i.e., F3, Fz, F4, C3, Cz, C4, P3, Pz and P4). The grand average signal from ROI_P300_ and traces for each level of origin, arousal and subjective significance are shown in Fig 5.

We observed the main effect of subjective significance (*F*(1.68, 56.96) = 8.64, *p* < .001, *η*_*p*_^*2*^ = 0.20). Mauchly’s test indicated that the assumption of sphericity had been violated for this factor (χ^2^ (2) = 0.81, *p* = .029), and therefore the degrees of freedom were corrected using Greenhouse-Geisser estimates of sphericity (∈ = 0.84). The post-hoc tests revealed that amplitude for highly significant words (*M* = 1.53, *SEM* = 0.55) was significantly more positive than for both low significant words (*M* = 0.94, *SEM* = 0.54; *t*(34) = 3.16, *p* = .007, *d* = 1.08) and medium significant words (*M* = 1.01, *SEM* = 0.52; *t*(34) = 3.95, *p* < .001, *d* = 1.35).

**Fig 6:**
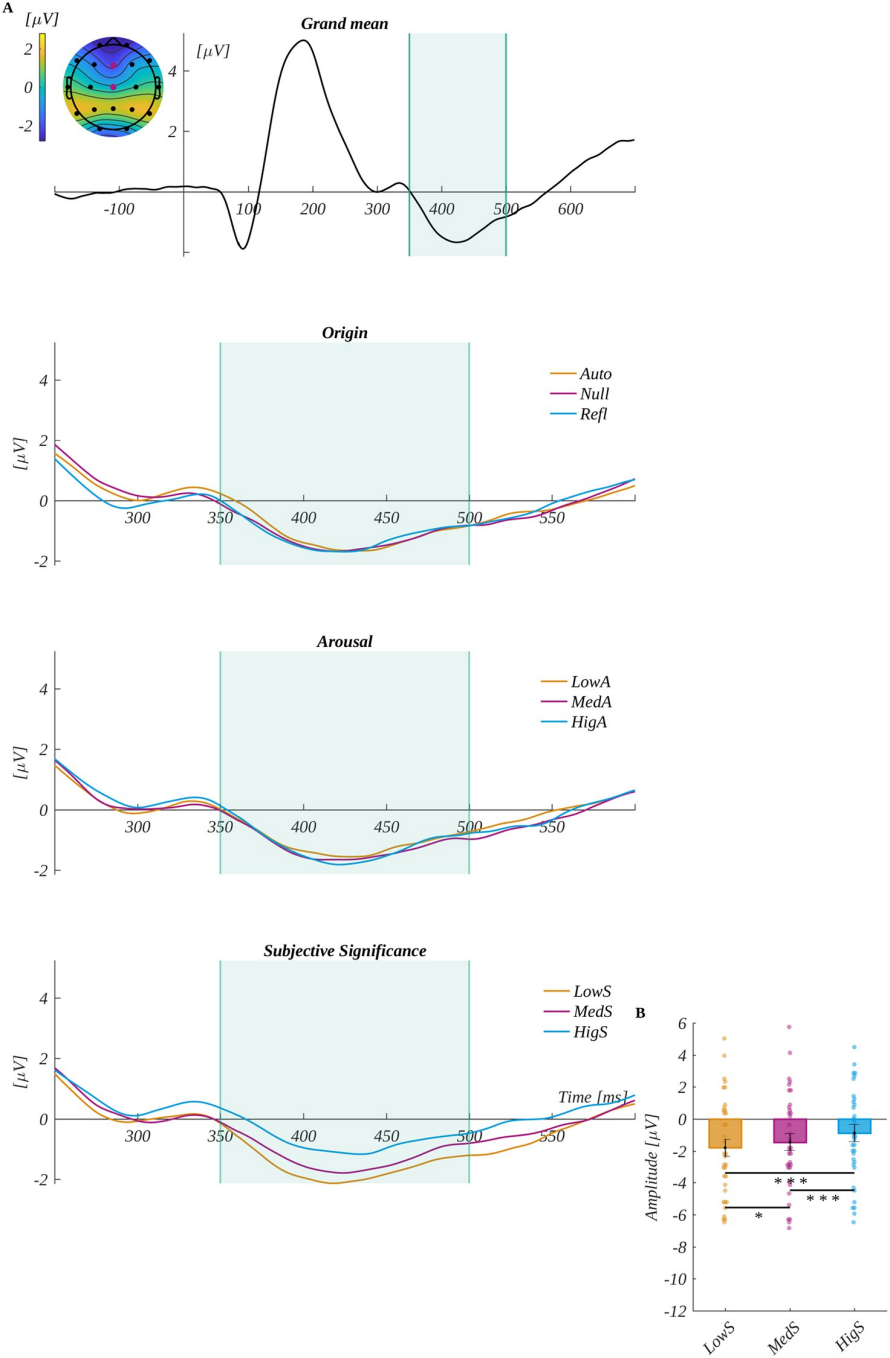
The N450 component results. The upper plot A is the grand average across electrodes from ROI_N450_. The marked time-interval was selected for the N450 analysis. The insert shows the topography of the mean potential from this period with the electrodes forming the ROI_N450_ marked with pink dots. The three bottom plots are the time courses of ERP averaged across electrodes from ROI_N450_ for each level of origin, arousal and subjective significance. B main effect for subjective significance. The bars represent the mean value, error bars SEM, individual dots mark the average amplitude of an individual subject in the given condition, the black horizontal lines indicate pairs of conditions with significantly different means (* *p* < .05, ** *p* < .01, *** *p* < .001).

We analysed the N450 component by averaging the ERP amplitude in the time window of 320–500 ms in the region of interest characteristic for N450 (ROIN450) (i.e., Cz and Pz). The grand mean from ROI N450 and mean for each level of origin, arousal and subjective significance are shown in Fig 6. The main effect of subjective significance was found (*F*(2, 68) = 22.242, *p* < .001, *η*_*p*_^*2*^ = 0.40). The post-hoc tests revealed that the amplitude was progressively less negative with the increase of subjective significance. Namely, low significant words (*M* = -1.79, *SEM* = 0.54) were significantly more negative than for both medium significant words (*M* = -1.45, *SEM* = 0.52; *t*(34) = -2.234, *p* = .032, *d* = 0.77) and highly significant words (*M* = -0.88, *SEM* = 0.55; *t*(34) = -6.312, *p* < .001, *d* = 2.16). Moreover, the amplitude for medium significant words was significantly more negative than for the highly significant words (*t*(34) = -5.078, *p* < .001, *d* = 1.74).

## Discussion

Considering behavioural data, we found that the highly significant words shorten the reaction time when compared to less significant words. This stands in contradiction to the previously found effects, where both the lower and the higher subjectively significant words were tied to shorter reaction times when compared to moderately subjectively significant stimuli [27, 58]. Both results may however support a preferential processing of highly subjectively significant stimuli. Based on just these behavioural results, doubts remain whether the subjective significance of the word was decoded fast enough to influence cognitive control, which would support the hypothesis that highly significant stimuli speed up conflict processing. Consequently, the behavioural effect of subjective significance in the current experiment should be perceived together with the EEG recordings that show more positive amplitudes for the high level of subjective significance throughout a few components (i.e., EPN, P300 and N450). It seems that highly significant words enhance the processing, thus making it faster.

On a behavioural level, we did not observe the interference caused by arousal, which could have been predicted based on the previous studies [27, 59–62]. In addition, no interaction of arousal and subjective significance was observed, We have reported on this particular interaction before [2], showing how high subjective significance reduces the interference from high arousal. The present study instead observed a main effect of subjective significance, strengthening the result, as this effect entails that significance increased inhibition effectiveness on all levels of arousal. As for the lack of an arousal effect, the alignment of the words on the dimension of valence may have narrowed the selection of highly arousing words that are usually tied to positive or negative concepts, which may be responsible for the obtained results. The behavioural effects seem to be highly dependent on the set of stimuli that are used, which reflects the actual language structure—highly positive and negative words are a natural part of this structure. Meanwhile, the lack of arousal effects appear to suggest that the proposed emotional dimensions of origin and subjective significance may be crucial for our understanding of the emotional interference in the EST, especially for not highly valenced words.

We also found that the reflective-originated verbal stimuli shorten the reaction time in EST when compared to words with no particular origin. This result is congruent with some of our previous EST [63]; where words of reflective origin elicited shorter reaction times than automatic words. However, it was not observed in other studies [33, 45], as these, contrary to the present study, manipulated valence. This is not the first time that origin effects were found to occur only when valence is controlled, as [43] also report origin ERP effects only for neutrally valenced words. The reflective emotional charge in words may subtly influence the processing speed in the absence of positive or negative emotional charge, which should be further explored.

In our ERP results, we observed significant changes in amplitude for both early and late components. First, we noted a decrease in P2 amplitude for reflective words when compared to words of no particular origin. This effect is especially interesting because it indicates a role for origin in very early word processing. Considering that the P2 component may be treated as a more sensitive measure of inhibitory control [37], it comes as no surprise that this difference was mirrored when we considered the behavioural results. These findings jointly indicate that processing words with an emotional load of reflective origin under conflict conditions is faster.

In EPN, we note a different pattern, again corresponding to a behavioural effect: subjective significance starts to shape the component’s amplitude such that the amplitude is more positive for highly significant words than for stimuli of both medium and low significance. This effect begins a series of effects where components’ characteristics for the EST, which were hitherto mostly found to be modulated by valence [31, 39–41], are significantly modulated by our emotional dimensions of interest. Origin now has a more complex role because it produces an interaction effect with arousal, such that for medium arousal, words with automatic origin result in a less positive amplitude than words with no particular origin. The key point here is that the other end of the origin scale (i.e., automatic origin) is also involved in modulating early components, which possibly indicates a temporal sequence in processing because its effects are seen after the signs of processing of reflective origin. It is interesting to note that although we scrupulously picked word stimuli in a way that would allow us to detect effects of arousal, we still observed no main effects of this factor. This can be seen as furthering the case that origin and significance may be emotional dimensions, which better explain the emotional interference seen in previous EST studies that did not control for them or investigate their effects. More broadly, in the context of the duality of mind perspective on emotion-cognition interaction of Imbir [26], a crucial result here is how early both the origin and subjective significance effects were observed. To modulate their respective ERPs, origin and significance of the word’s emotional load had to be decoded already at 150 ms and 230 ms respectively. This supports the postulates of the theory: first, that subjective significance and origin are emotional dimensions that influence early processing of emotional stimuli; second, that there exists a preferential processing of highly significant stimuli, which contrary to most previously reported emotion-cognition interactions, is an increase of inhibition control effectiveness for highly significant stimuli. To some extent, a similar increase in effectiveness can be seen for reflective stimuli, although as we have seen, only in the absence of high valence.

For the later ERP components, we observed effects of subjective significance for both P300 and N450, which would put our results in line with some previous studies [64]. For P300—related to the stimuli categorisation and, in EST, to the emotionality of stimuli—there was an effect of significantly more positive amplitude for the words of high significance in comparison with stimuli of low and medium significance. This result shows the influence of the dimension of subjective significance on the processing of words, which is unrelated to the dimensions of arousal or valence that are usually correlated with it [64].

For N450—a component generally associated with processing the conflict and, in the EST paradigm, the incongruence in stimuli [47]—there was a significant effect of gradually less negative amplitude along with the increasing level of subjective significance. This effect is similar to the one obtained previously [27]. This can be understood as a reflection of conceptual processing, which is especially present when detecting stimuli of low significance (and, by that, further deciding to omit such stimuli due to their low relevance to one’s goals). Therefore, this result supports our hypothesis and it shows the gradual improvement in cognitive control (fewer negative amplitudes). We did not find any effect for origin or arousal in these late-stimuli processing sections. However, it is also worth mentioning that there is a visible effect of the subjective significance having higher amplitude across all of the components in our results (despite the localisation differences between electrodes). This might be suggested as a future direction for further studies.

This study has observed several limitations. Considering the design, all of the dimensions describing emotions in the current study were crossed orthogonally, which does not reflect a naturally occurring relation between variables [2] and thus may produce some misleading effects. However, this is the only way to get to know how each dimension influences cognitive control. In addition, the design that we used enables us to control word-related factors, such as subjective significance, word length or the frequency of use potentially influencing results. However, the procedure was also quite complex and cognitively costly. Due to a large number of trials, we might expect fatigue to grow with subsequent tasks. To avoid this type of contamination, both trials and groups of words were fully randomised. The final limitation refers to the sample. The subjects only included students. Therefore, the sample was homogenous and narrowed to a specific group. However, testing specific groups allowed us to eliminate some confounding factors.

In conclusion, we would like to highlight that the current experiment showed an effect that can be surprising on a basic level of understanding of cognitive control interaction with activational processes (typically accompanying emotions but analysed here in neutral stimuli). We demonstrated the increased effectiveness of control when the subjective significance level was high and when the stimulus’s origin was reflective. However, the typically pattern that we investigated is the opposite: impairment is associated with increased arousal levels, and for primary emotions, which can be identified as automatically originated [22]. The subjective significance and reflective origin represent the measures characterising the second-order emotional processes [19, 21, 24, 25] that engage verbalisation and reflective thinking to provide the interpretation and understanding of environmental stimuli. The engagement of deliberation provokes different types of activation mechanisms. In most cases, reflective processes are thought to be too subjective and thus immeasurable in a systematic way. Our study showed that with precise control of other factors, the reflective processes could be operationalised and they can also improve cognitive control effectiveness.

## Supporting information

S1 Appendix S1 Appendix contain all of the 405 words that we used in the experiment, their affective measures, length and frequency of use, as well as full analyses showing validity of the selected stimuli. (XLSX)
